# Differences in tumor-infiltrating lymphocyte density and prognostic factors for breast cancer by patient age

**DOI:** 10.1186/s12957-022-02513-5

**Published:** 2022-02-17

**Authors:** Koji Takada, Shinichiro Kashiwagi, Yuka Asano, Wataru Goto, Tamami Morisaki, Masatsune Shibutani, Hiroaki Tanaka, Kosei Hirakawa, Masaichi Ohira

**Affiliations:** 1 Department of Breast and Endocrine Surgery, Osaka City University Graduate School of Medicine, 1-4-3 Asahi-machi, Abeno-ku, Osaka, 545-8585 Japan; 2 Department of Gastroenterological Surgery, Osaka City University Graduate School of Medicine, 1-4-3 Asahi-machi, Abeno-ku, Osaka, 545-8585 Japan

**Keywords:** Breast cancer, Tumor-infiltrating lymphocytes, Tumor-immune microenvironment, Age, Preoperative chemotherapy

## Abstract

**Background:**

Lymphocytes that surround cancer participate in tumor-related immune responses and are called tumor-infiltrating lymphocytes (TILs). Several recent reports suggest TILs index the tumor microenvironment and predict the therapeutic effect of chemotherapy. However, only few studies have studied the relationship between age and TILs. Aging reduces host immunity, and we predict that it may also affect TILs. Thus, we hypothesized that older breast cancer (BC) patients may have low TIL density than younger BC patients. Here, we retrospectively analyzed the differences in TILs by age and the therapeutic effects of preoperative chemotherapy (POC) in BC patients who were aged either less than 45 years or more than 60 years.

**Methods:**

We retrospectively examined the data of 356 breast cancer patients who underwent POC, including 75 patients aged ≤ 45 years and 116 patients aged > 60 years. Using pre-treatment needle biopsy specimens, TIL density was compared for each age group by Student’s *t*-test. After analyzing different factors that affect TIL density, prognostic factors were also examined.

**Results:**

Older patients with triple-negative BC had significantly lower TIL density than younger patients, while in human epidermal growth factor receptor 2 (HER2)-enriched BC, TIL density was significantly higher in the younger age group than that in the older age group. In addition, younger patients with HER2-rich breast cancer showed significantly higher complete pathological response rates than older patients with HER2-rich BC. In addition, significant differences in overall survival were observed among these patients with triple-negative BC.

**Conclusions:**

Our study suggests that younger BC patients possess significantly higher TIL density than older patients. These differences may influence the therapeutic efficacy in highly immunogenic subtypes.

**Supplementary Information:**

The online version contains supplementary material available at 10.1186/s12957-022-02513-5.

## Background

Tumor-infiltrating lymphocytes (TILs) surround cancer tissue and are involved in tumor-related immune responses [1]. Moreover, as components of the tumor microenvironment (TME), TILs allow the prediction of the therapeutic efficacy of chemotherapy [2–4]. In patients with breast cancer (BC), an increase in TIL density correlated with an increase in the pathologic complete response (pCR) rate, along with improved disease-free survival (DFS) and overall survival (OS) [5, 6]. Further, the TIL density in breast cancer differs depending on the subtype. For instance, hormone receptor-negative breast cancers (HR-BC), such as triple-negative breast cancer (TNBC) and human epidermal growth factor receptor 2-enriched breast cancer (HER2-enriched BC), show high TIL density [7–9]. However, there are fewer reports on factors other than BC subtypes that affect the TIL density.

Currently, the standard treatment is based on the results of various clinical trials. For instance, some clinical trials suggest the prognosis and treatment effect differ depending on the age of the patients [10–12], and several pooled studies have reported differences in the treatment effect due to age [5, 13, 14]. However, until now, only few studies have assessed the relationship between age and TIL density. While increased age may reduce host immunity [15], we can also hypothesize that it affects TIL density. Moreover, clinical trials on the association of TILs and therapeutic effects have not correlated age and TILs [12, 16–19], and most of them have stratified patients into two groups based on TILs or age and performed only *t*-test analyses to compare the groups.

First, we decided to compare TIL density for each age group, and if the TIL density decreased with age, we hypothesized that omitting the middle-aged group would polarize the younger and the older age groups. We also tested the hypothesis that the therapeutic effect and prognoses of patients may differ with TIL density. Thus, here, we retrospectively analyzed the differences in TIL density by age and analyzed the therapeutic effects in patients with BC ≤ 45 years or > 60 years of age who were treated with preoperative chemotherapy (POC).

## Methods

### Patient background

A total of 356 patients with BC received POC between February 2007 and March 2018 at the Osaka City University Hospital, Japan, and were retrospectively recruited in the study. Further, we compared the TIL density in patients aged ≤ 45 years (younger group, *n* = 75) versus those aged > 60 years (older group, *n* = 116). The patients were pathologically diagnosed with BC using core needle biopsy (CNB) or vacuum-assisted biopsy (VAB), and by immunohistochemical staining of the specimen to evaluate the expression of estrogen receptor (ER), progesterone receptor (PgR), HER2, and Ki67. Based on the results, the subtypes were classified as follows: HER2-enriched BC (ER−, PgR−, and HER2+); TNBC (ER−, PgR−, and HER2−); HR+HER2+BC (ER+ and/or PgR+, and HER2+); and HR+HER2-BC (ER+ and/or PgR+, and HER2−). Before chemotherapy, the staging of BC was evaluated using ultrasonography (US), computed tomography (CT), and bone scintigraphy. POC was administered in BC patients diagnosed with stage IIA (T1, N1, M0 or T2, N0, M0), IIB (T2, N1, M0 or T3, N0, M0), IIIA (T1–2, N2, M0 or T3, N1–2, M0), IIIB (T4, N0–2, M0), or IIIC (T1–4, N3, M0). The POC regimen was comprised of four courses of FEC100 (500 mg/m^2^ fluorouracil, 100 mg/m^2^ epirubicin, and 500 mg/m^2^ cyclophosphamide) every 3 weeks, followed by 12 courses of 80 mg/m^2^ paclitaxel administered weekly. For HER2+ BC patients, an additional weekly (2 mg/kg) or tri-weekly (6 mg/kg) dosage of trastuzumab was administered during paclitaxel treatment [20–22]. The anti-tumor effects of POC were evaluated according to the Response Evaluation Criteria in Solid Tumors [23]. Further, patients with clinical partial response (cPR) and complete response (cCR) were defined as “responders” in the objective response rate (ORR), whereas patients with clinical stable disease (cSD) and clinical progressive disease (cPD) were defined as “non-responders.” After POC, all the patients underwent mastectomy or breast-conserving surgery [24]. A pathologic complete response (pCR) was defined as the complete disappearance of the invasive components of the lesion with or without intraductal components, including that in the lymph nodes according to the National Surgical Adjuvant Breast and Bowel Project B-18 protocol [25].

Post-surgery, standard adjuvant therapy was administered according to each subtype and surgical procedure. During adjuvant therapy, all the patients were evaluated for tumor recurrence by physical examination, US, and CT and bone scintigraphy every 3, 6, and 12 months, respectively. The median follow-up time was 1281 days (range, 13–3675 days) after surgery.

### Histopathological evaluation of TIL density

TIL density was evaluated using pretreatment specimens obtained by CNB or VAB. The TILs were defined and evaluated based on the International TILs Working Group 2014 [1] as the average of the infiltrating lymphocytes within the tumor stroma at five randomly selected fields. Next, the results were classified into four classes (3: > 50%; 2: > 10–50%; 1: ≤ 10%; or 0: absent) (Supplementary Fig. S1). Further, we defined scores 2 and 3 as “high,”, and scores 1 and 0 as “low” according to previous reports [26, 27]. Thus, in brief, the cut-off value of TIL density was set to 10%.

### Statistical analysis

All statistical analyses were performed using the JMP software package (SAS, Tokyo, Japan). The distribution of TIL density by age was evaluated using Student’s *t*-test. Pearson’s chi-square test was used to evaluate the relationship between each categorical variable. Prognostic analyses, such as DFS or OS, were examined using the Kaplan–Meier method and log-rank test. The hazard ratio (HR) and 95% confidence interval (CIs) were calculated using the Cox proportional hazards model. Multivariable analysis was performed using the Cox regression model. A *P*-value < 0.05 was considered statistically significant.

## Results

### Clinicopathological features of BC patients

The clinicopathological features of patients (*n* = 356) treated with POC have been summarized in Table 1. The patients were operated on at a median age of 55 years (range, 24–78 years) and the median tumor diameter was 28.7 mm (range, 9.2–119.8 mm). Skin infiltration was observed in 58 patients (16.3%). Further, imaging methods of diagnosis did not indicate lymph node metastasis in 121 patients (34.0 %). The number of ER-negative, PgR-negative, and HER2-positive patients was 187 (52.5 %), 242 (68.0 %), and 125 (35.1 %), respectively. Moreover, Ki67-high (above 14%) was observed in 239 patients (67.1 %). Based on these results, the BC subtypes were classified as follows—HR+HER2−: 126 patients (35.4 %), HR+HER2+: 47 patients (13.2 %), HER2-enriched: 78 patients (21.9 %), and TNBC: 105 patients (29.5 %). Furthermore, the responders for ORR reached 88.8%, the rate of pCR post-operative pathology was 33.1%, and 161 patients (45.2%) showed high TIL density.

**Table 1:** Clinicopathological features of 356 patients who were treated with preoperative chemotherapy

Parameters	All patients (*n* = 356) (%)	Younger (*n* = 75) (%)	Elderly (*n* = 116) (%)
Age (years)	55 (24–78)	41 (24–45)	67 (61–78)
Tumor size (mm)	28.7 (9.2–119.8)	29.5 (9.9–82.6)	27.3 (9.2–89.8)
Skin infiltration			
Negative/positive	298 (83.7%)/58 (16.3%)	68 (90.7%)/7 (9.3%)	90 (77.6%)/26 (22.4%)
Lymph node metastasis			
N0/N1/N2/N3	121 (33.9%)/133 (37.4%)/68 (19.1%)/34(9.6%)	28 (37.3%)/28 (37.3%)/14 (18.7%)/5 (6.7%)	44 (37.9%)/36 (31.0%)/22 (19.0%)/14(12.1%)
Estrogen receptor			
Negative/positive	187 (52.5%)/169 (47.5%)	37 (49.3%)/38 (50.7%)	67 (57.8%)/49 (42.2%)
Progesterone receptor			
Negative/positive	242 (68.0%)/114 (32.0%)	42 (56.0%)/33 (44.0%)	89 (76.7%)/27 (23.3%)
HER2			
Negative/positive	231 (64.9%)/125 (35.1%)	47 (62.7%)/28 (37.3%)	69 (59.5%)/47 (40.5%)
Ki67			
≤ 14 %/> 14 %	117 (32.9%)/239 (67.1%)	22 (29.3%)/53 (70.7%)	40 (34.5%)/76 (65.5%)
Intrinsic subtype			
HR+HER2-BC/HR+HER2+BC/HER2BC/TNBC	126 (35.4%)/47 (13.2%)/78 (21.9%)/105 (29.5%)	24 (32.0%)/16 (21.3%)/12 (16.0%)/23 (30.7%)	39 (33.6%)/11 (9.5%)/36 (31.0%)/30 (25.9%)
Objective response rate			
Non-responders/responders	40 (11.2%)/316 (88.8%)	5 (6.7%)/70 (93.3%)	17 (14.7%)/99 (85.3%)
Pathological response			
Non-pCR/pCR	238 (66.9%)/118 (33.1%)	46 (61.3%)/29 (38.7%)	78 (67.2%)/38 (32.8%)
TILs			
Low/high	195 (54.5%)/161 (45.2%)	31 (41.3%)/44 (58.7%)	65 (56.0%)/51 (44.0%)

*HER* human epidermal growth factor receptor, *CR* complete response, *TILs* tumor-infiltrating lymphocytes

Further, while most of the clinicopathological factors were not significantly different, the rate of skin infiltration and PgR-negative status were significantly higher in the older than in the younger patients (*P* = 0.002 and *P* = 0.003, respectively) (Table 2). Moreover, the ORR, although statistically insignificant, was found to be higher in the younger than in the older patients (*P* = 0.091).

**Table 2:** Difference in clinicopathological features due to TILs in younger and elderly patients

Parameters	Tumor-infiltrating lymphocytes (*n* = 191)		
	Low (*n* = 96)	High (*n* = 95)	*p* value
Age (years)			
≤ 45	31 (32.3%)	44 (46.3%)	0.047
> 60	65 (67.7%)	51 (53.7%)	
Tumor size (mm)			
≤ 20.0	20 (20.8%)	14 (14.7%)	0.271
> 20.0	76 (79.2%)	81 (85.3%)	
Skin infiltration			
Negative	71 (74.0%)	87 (91.6%)	0.001
Positive	25 (26.0%)	8 (8.4%)	
Lymph node status			
Negative	33 (34.4%)	39 (41.1%)	0.341
Positive	63 (65.6%)	56 (58.9%)	
Estrogen receptor			
Negative	37 (38.5%)	67 (70.5%)	< 0.001
Positive	59 (61.5%)	28 (29.5%)	
Progesterone receptor			
Negative	55 (57.3%)	76 (80.0%)	0.001
Positive	41 (42.7%)	19 (20.0%)	
Hormone receptor			
Negative	35 (36.5%)	66 (69.5%)	< 0.001
Positive	61 (63.5%)	29 (30.5%)	
HER2			
Negative	69 (71.9%)	47 (49.5%)	0.002
Positive	27 (28.1%)	48 (50.5%)	
Ki67			
≤ 14%	37 (38.5%)	25 (26.3%)	0.071
> 14%	59 (61.5%)	70 (73.7%)	
ORR			
Non-responders	18 (18.8%)	4 (4.2%)	0.002
Responders	78 (81.2%)	91 (95.8%)	
Pathological response			
Non-pCR	79 (82.3%)	45 (47.4%)	< 0.001
pCR	17 (17.7%)	50 (52.6%)	

*TILs* tumor-infiltrating lymphocytes *HER*, human epidermal growth factor receptor, *ORR* objective response rate, *CR* complete response

### Correlation of TIL density with clinicopathological features and prognosis of patients

First, the 356 patients were divided into high and low TIL density groups, and their correlation with clinicopathological factors was examined (Supplementary Table S1). The following characteristics were significantly different between the low TILs and high TILs group: ≥ 45 years (*P* = 0.008), skin invasion (*P* = 0.001), ER-positive (*P* < 0.001), PgR-positive (*P* < 0.001), HER2-negative (*P* = 0.011), Ki67-high (*P* < 0.001), low ORR (*P* = 0.001), and low pCR rate (*P* < 0.001).

Further, the high TIL density group showed significantly better DFS than the low TIL density group in HER2-enriched (*P* = 0.012) and TNBC (*P* = 0.002) categories (Supplementary Fig. S2). Therefore, DFS was better in the high TIL density group despite no significant difference in HR+ BC (*P* = 0.011). However, the high TIL density group had better OS, although not statistically significant, than the low TIL density group in TNBC category (*P* = 0.057, log-rank), but there was no significant difference between the difference of TIL density (Supplementary Fig. S3). Further, in the univariate analysis for DFS, the high TIL density group was associated with significantly better DFS (*P* = 0.010, HR = 0.512) than the low TIL density group (Supplementary Table S2). However, in the multivariate analysis, TIL density was not an significant independent factor for DFS (*P* = 0.227, HR = 0.699) and since skin invasion (*P* = 0.012, HR = 2.180), lymph node metastasis (*P* = 0.001, HR = 2.918), HER2-positive (*P* = 0.020, HR = 0.498), responders in ORR (*P* < 0.001, HR = 0.247), and pCR (*P* < 0.001, HR = 0.315) influenced the DFS. Additionally, difference in OS due to TILs was insignificant even in the univariate analysis (*P*=0.214, HR = 0.660) (Supplementary Table S3).

**Fig. 1:**
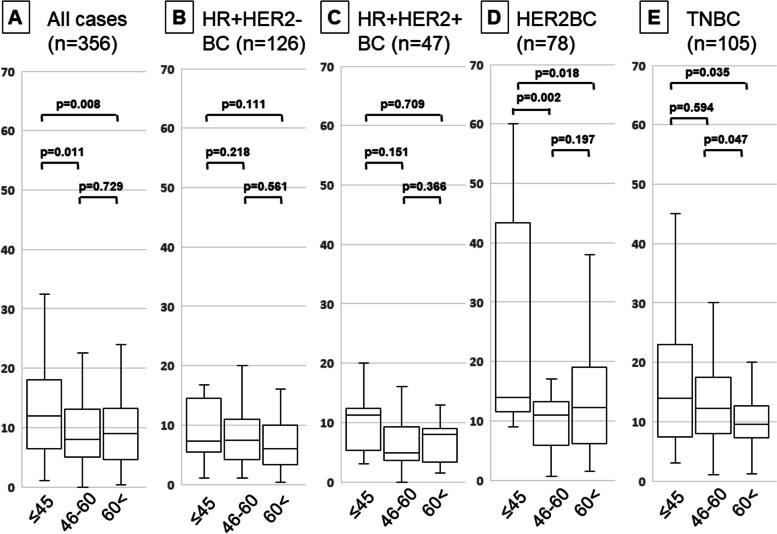
Correlation of TIL density with age of BC patients. Patients were grouped based on their BC subtype as: **a**) all cases, **b**) HR+HER2-, **c**) HR+HER2+, **d**) HER2-enriched, and **e**) TNBC. The TIL density in each age group in each subtype has been indicated using box-plot distribution analysis. *P*-values in the figure indicate statistical significance for each comparison obtained using *t*-test

Further, the patients were classified based on age as ≤ 45 years, 46–60 years, and ≥ 61 years, and the distribution of TIL density was analyzed using a *t*-test (Fig. 1). Our analysis did not indicate a significant difference in HR+ BC for any of the age groups. However, for HER2-enriched BC, patients aged ≤ 45 years had significantly higher TIL density than patients in the other age groups (vs. 46–60 years: *P* = 0.002, and vs. ≥ 61 years: *P* = 0.018). Furthermore, in the TNBC category, the patients aged ≥ 61 years had significantly higher TIL density than patients in other age groups (vs. ≤ 40 years: *P* = 0.035, and vs. 46–60 years: *P* = 0.047).

### Examination of clinicopathological factors and prognosis in the younger and older BC patients

First, we studied the correlation between TIL density and clinicopathological factors in the younger and older patients (Table 2). Although patients aged 46–60 years were excluded from the analysis, the characteristics of the high TIL density group were similar to those for all patients: > 60 years (*P* = 0.047), skin infiltration (*P* = 0.001), ER-positive (*P* < 0.001), PgR-positive (*P* = 0.001), HER2-negative (*P* = 0.002), lower ORR (*P* = 0.002), and lower pCR rate (*P* < 0.001).

Further, younger patients showed significantly higher pCR rates than older patients in the HR+HER2- and HER2-enriched BC category (*P* = 0.021 and *P* = 0.048, respectively) (Table 3). Moreover, in HR+HER2+BC, the responder rate for ORR was significantly higher in the younger patients than in the older patients (*P* = 0.009). However, no significant difference was observed in the effect of POC on TNBC.

**Table 3:** Difference in clinicopathological features due to age

Parameters	All intrinsic subtype (*n* = 191)			HR+HER2-BC (*n* =61)			HR+HER2+BC (*n* =27)			HER2BC (*n* =48)			TNBC (*n* =53)		
	Young (*n* =75)	Elderly (*n* = 116)	*p* value	Young (*n* =24)	Elderly (*n* = 39)	*p* value	Young (*n* =16)	Elderly (*n* = 11)	*p* value	Young (*n* =12)	Elderly (*n* = 36)	*p* value	Young (*n* =23)	Elderly (*n* = 30)	*p* value
Tumor size (mm)															
≤ 20.0	10 (13.3%)	24 (20.7%)	0.194	2 (8.3%)	7 (17.9%)	0.290	3 (18.8%)	2 (18.2%)	0.970	2 (16.7%)	7 (19.4%)	0.831	3 (13.0%)	8 (26.7%)	0.225
> 20.0	65 (86.7%)	92 (79.3%)		22 (91.7%)	32 (82.1%)		13 (81.2%)	9 (81.8%)		10 (83.3%)	29 (80.6%)		20 (87.0%)	22 (73.3%)	
Skin infiltration															
Negative	68 (90.7%)	90 (77.6%)	0.020	20 (83.3%)	29 (74.4%)	0.405	14 (87.5%)	6 (54.5%)	0.055	12 (100.0%)	29 (80.6%)	0.098	22 (95.7%)	26 (86.7%)	0.267
Positive	7 (9.3%)	26 (22.4%)		4 (16.7%)	10 (25.6%)		2 (12.5%)	5 (45.5%)		0 (0.0%)	7 (19.4%)		1 (4.3%)	4 (13.3%)	
Lymph node status															
Negative	28 (37.3%)	44 (37.9%)	0.934	8 (33.3%)	12 (30.8%)	0.832	9 (56.2%)	2 (18.2%)	0.048	4 (33.3%)	17 (47.2%)	0.401	7 (30.4%)	13 (43.3%)	0.337
Positive	47 (62.7%)	72 (62.1%)		16 (66.7%)	27 (69.2%)		7 (43.8%)	9 (81.8%)		8 (66.7%)	19 (52.8/%)		16 (69.6%)	17 (56.7%)	
Estrogen receptor															
Negative	37 (49.3%)	67 (57.8%)	0.254	2 (8.3%)	0 (0.0%)	0.067	0 (0.0%)	1 (9.1%)	0.219	–	–		–	–	
Positive	38 (50.7%)	49 (42.2%)		22 (91.7%)	39 (100.0%)		16 (100.0%)	10 (90.9%)		–	–		–	–	
Progesterone receptor															
Negative	42 (56.0%)	89 (76.7%)	0.003	5 (20.8%)	16 (41.0%)	0.099	2 (12.5%)	7 (63.6%)	0.006	–	–		–	–	
Positive	33 (44.0%)	27 (23.3%)		19 (79.2%)	23 (59.0%)		14 (87.5%)	4 (36.4%)		–	–		–	–	
Hormone receptor-															
Negative	35 (46.7%)	66 (56.9%)	0.167	–	–		–	–		–	–		–	–	
Positive	40 (53.3%)	50 (43.1%)		–	–		–	–		–	–		–	–	
HER2															
Negative	47 (62.7%)	69 (59.5%)	0.660	–	–		–	–		–	–		–	–	
Positive	28 (37.3%)	47 (40.5%)		–	–		–	–		–	–		–	–	
Ki67															
≤ 14%	22 (29.3%)	40 (34.5%)	0.458	12 (50.0%)	21 (53.8%)	0.767	7 (43.8%)	2 (18.2%)	0.166	1 (8.3%)	12 (33.3%)	0.091	2 (8.7%)	5 (16.7%)	0.396
> 14%	53 (70.7%)	76 (65.5%)		12 (50.0%)	18 (46.2%)		9 (56.2%)	9 (81.8%)		11 (91.7%)	24 (66.7%)		21 (91.3%)	25 (83.3%)	
ORR															
Non-responders	5 (6.7%)	17 (14.8%)	0.091	2 (8.3%)	8 (20.5%)	0.199	0 (0.0%)	4 (36.4%)	0.009	0 (0.0%)	1 (2.8%)	0.560	3 (13.0%)	4 (13.3%)	0.975
Responders	70 (93.3%)	99 (85.2%)		22 (91.7%)	31 (79.5%)		16 (100.0%)	7 (63.6%)		12 (100.0%)	35 (97.2%)		20 (87.0%)	26 (86.7%)	
Pathological response															
Non-pCR	46 (61.3%)	78 (67.2%)	0.403	18 (75.0%)	37 (94.9%)	0.021	13 (81.2%)	10 (90.9%)	0.488	1 (8.3%)	14 (38.9%)	0.048	14 (60.9%)	17 (56.7%)	0.758
pCR	29 (38.7%)	38 (32.8%)		6 (25.0%)	2 (5.1%)		3 (18.8%)	1 (9.1%)		11 (91.7%)	22 (61.1%)		9 (39.1%)	13 (43.3%)	
TILs															
Low	31 (41.3%)	65 (56.0%)	0.047	14 (58.3%)	31 (79.5%)	0.071	7 (43.8%)	9 (81.8%)	0.048	1 (8.3%)	10 (27.8%)	0.165	9 (39.1%)	15 (50.0%)	0.431
High	44 (58.7%)	51 (44.0%)		10 (41.7%)	8 (20.5%)		9 (56.2%)	2 (18.2%)		11 (91.7%)	26 (72.2%)		14 (60.9%)	15 (50.0%)	

*HER* human epidermal growth factor receptor, *ORR* objective response rate, *CR* complete response, *TILs* tumor-infiltrating lymphocytes

**Fig. 2:**
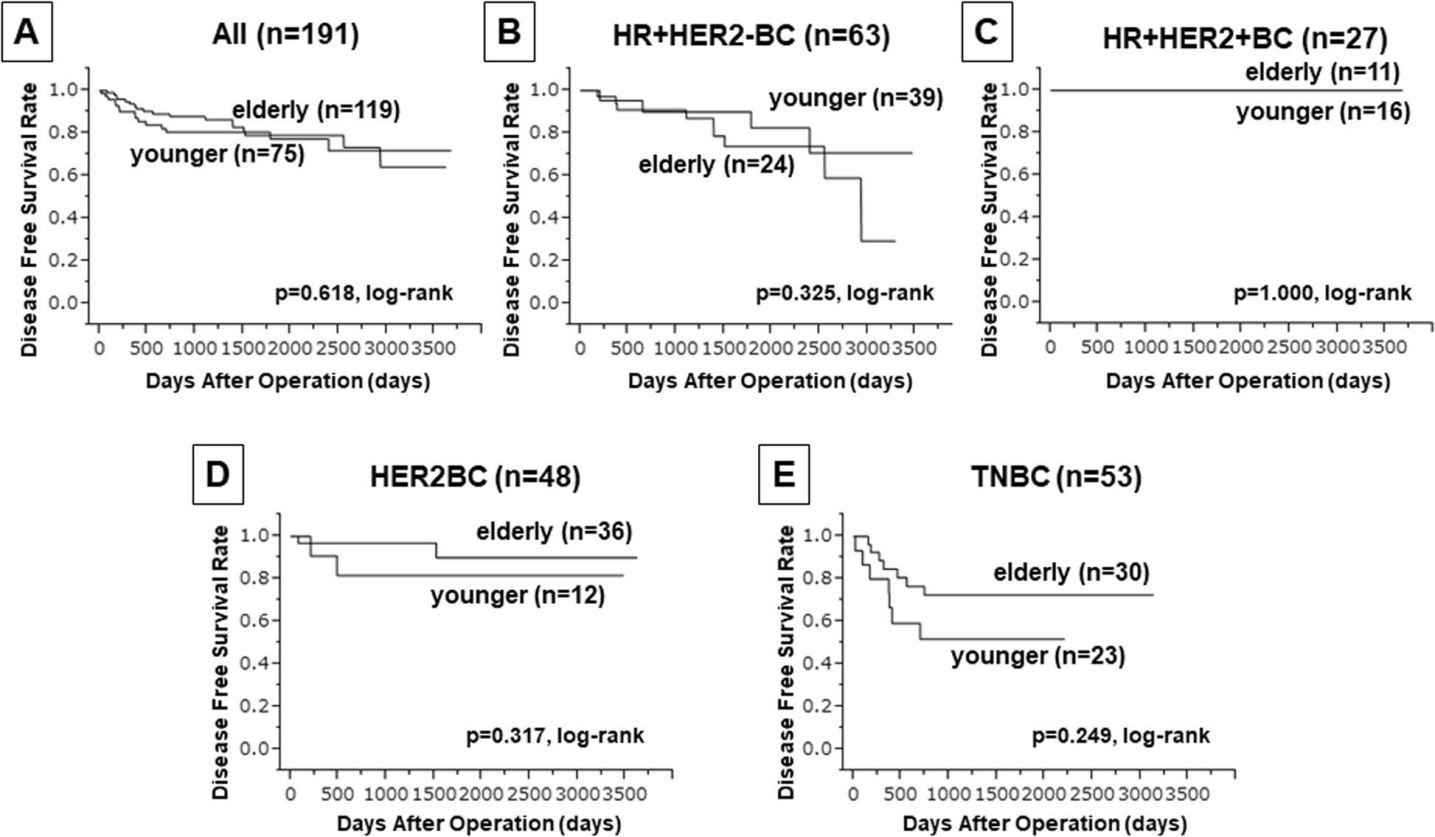
Comparison of disease-free survival (DFS) between younger and older patients with varied BC subtypes. Kaplan-Meier DFS analysis has been indicated for patients grouped based on their BC subtype as: **a**) all cases, **b**) HR+HER2-, **c**) HR+HER2+, **d**) HER2-enriched, and **e**) TNBC. *P*-values in the figure indicate statistical significance for each comparison obtained using log-rank test

**Fig. 3:**
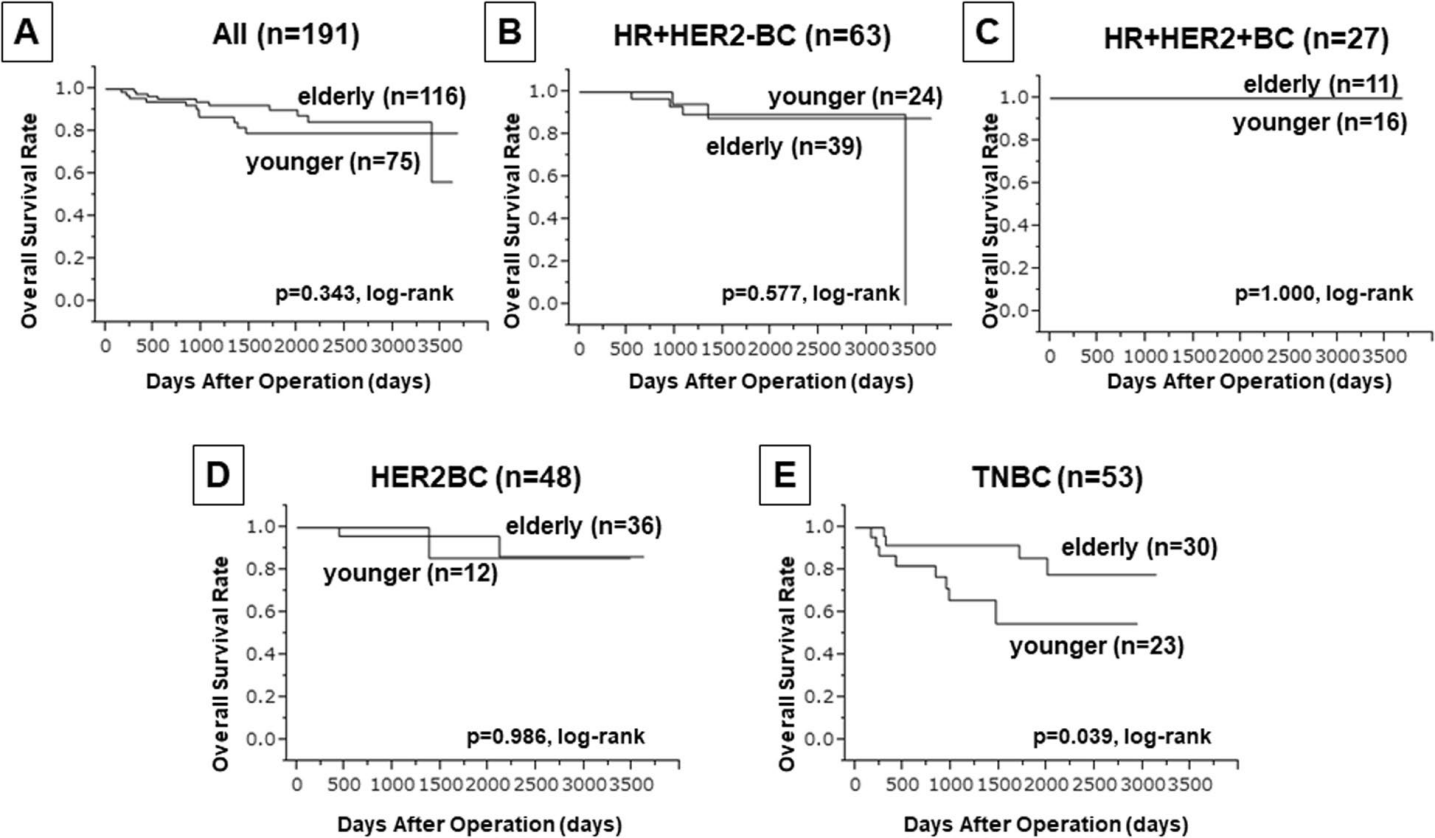
Comparison of overall survival (OS) between younger and older patients with varied BC subtypes. Kaplan-Meier OS analysis has been indicated for patients grouped based on their BC subtype as: **a**) all cases, **b**) HR+HER2-, **c**) HR+HER2+, **d**) HER2-enriched, and **e**) TNBC. *P*-values in the figure indicate statistical significance for each comparison obtained using log-rank test

Next, when DFS was compared between the younger and older patients, no significant difference was found overall or in any subtype (Fig. 2). Moreover, our analysis indicated that age or TILs was not a predictor of DFS in the univariate analysis (*P* = 0.619 and *P* = 0.066, respectively) (Table 4). Although upon comparison of OS, a significant difference was observed between younger and older patients with TNBC (*P* = 0.039, log-rank) (Fig. 3), the results were contrasting and suggested better OS in older patients than in younger patients. Additionally, in univariate analysis with OS, no significant difference in age and TIL density was observed (*P* = 0.346 and *P* = 0.216, respectively) (Table 5).

**Table 4:** Univariate and multivariate analysis with respect to DFS in younger and elderly patients

Parameters	Univarite analysis			Multivariate analysis		
	Hazard ratio	95% CI	*p* value	Hazard ratio	95% CI	*p* value
Age at operation (years)						
≤ 45 vs > 60	0.916	0.651–1.300	0.619			
Tumor size (mm)						
≤ 20 vs > 20	0.674	0.309–1.684	0.373			
Skin infiltration						
Negative vs positive	2.629	1.140–5.582	0.025	2.597	1.075–5.858	0.035
Lymph node status						
Negative vs positive	4.935	1.756–20.600	0.001	3.981	1.385–16.828	0.008
Estrogen receptor						
Negative vs positive	0.738	0.358–1.469	0.390			
Progesterone receptor						
Negative vs positive	0.733	0.322–1.524	0.418			
Hormone receptor						
Negative vs positive	0.675	0.327–1.344	0.265			
HER2						
Negative vs positive	0.237	0.070–0.602	0.001	0.479	0.130–1.423	0.193
Intrinsic subtype						
Not TNBC vs TNBC	2.710	1.356–5.392	0.005	2.418	1.080–5.456	0.032
Ki67						
≤ 14% vs > 14%	2.339	1.066–5.872	0.033	2.489	1.089–6.417	0.030
Objective response rate						
Non-responders vs responders	0.309	0.145–0.734	0.010	0.381	0.159–0.984	0.047
Pathological response						
Non-pCR vs pCR	0.195	0.058–0.499	< 0.001	0.238	0.065–0.685	0.006
TILs						
Low vs high	0.523	0.253–1.045	0.066	0.991	0.431–2.231	0.982

*DFS* disease-free survival, *CI* confidence intervals, *HER* human epidermal growth factor receptor, *pCR* pathological complete response, *TILs* tumor-infiltrating lymphocytes

**Table 5:** Univariate and multivariate analysis with respect to OS in younger and elderly patients

Parameters	Univarite analysis			Multivariate analysis		
	Hazard ratio	95% CI	*p* value	Hazard ratio	95% CI	*p* value
Age at opetation (years)						
≤ 45 vs > 60	0.813	0.524–1.255	0.346			
Tumor size (mm)						
≤ 20 vs > 20	1.188	0.402–5.074	0.778			
Skin infiltration						
Negative vs positive	5.034	1.940–12.433	0.002	6.899	2.467–18.908	< 0.001
Lymph node status						
Negative vs positive	4.239	1.227–26.631	0.019	2.999	0.815–19.389	0.106
Estrogen receptor						
Negative vs positive	0.474	0.169–1.167	0.107			
Progesterone receptor						
Negative vs positive	0.475	0.137–1.285	0.151			
Hormone receptor						
Negative vs positive	0.441	0.157–1.085	0.076			
HER2						
Negative vs positive	0.283	0.066–0.844	0.021	0.721	0.149–2.809	0.645
Intrinsic subtype						
Not TNBC vs TNBC	3.966	1.640–10.130	0.002	3.703	1.323–11.575	0.012
Ki67						
≤ 14% vs >14%	2.730	1.004–9.518	0.049	2.271	0.768–8.314	0.144
Objective response rate						
Non-responders vs responders	0.244	0.097–0.692	0.010	0.259	0.090–0.797	0.020
Pathological response						
Non-pCR vs pCR	0.241	0.056–0.718	0.009	0.384	0.082–1.332	0.137
TILs						
Low vs high	0.578	0.232–1.380	0.216			

*OS* overall survival, *CI* confidence intervals, *HER* human epidermal growth factor receptor, *pCR* pathological complete response, *TILs* tumor-infiltrating lymphocytes

## Discussion

The characteristics of BC in the older patients have been often reported. For example, large tumor size [13, 28–30], frequent skin infiltration [29, 31], infrequent lymph node metastasis [28, 30], high rate of HR positivity [13, 28], and fewer HER2-positive tumors [28–30] have been reported in older patients. The clinicopathological characteristics of older BC patients in our study show a strong correlation to the decision of administering POC or not, though some features similar to those reported by others were identified.

While age-related differences in pCR rates have not been reported in several clinical trials, a pooled analysis observed a high pCR rate in younger BC patients [14]. Moreover, reports suggest that the pCR rate decreased with age [10, 13]. Analysis of BC based on subtype in these studies suggested a strong correlation between HR+HER2- and TNBC, whereas no significant difference with age was observed in HER2-positive BC, which differed in our study, and the exact reason remains to be identified. Further, there are various molecular subtypes of TNBC, and the age at onset and pCR rates differ across studies [32–34]. We anticipate that our analysis may have been affected by differences in molecular subtypes of TNBC or due to differences in the chemotherapy regimen. Furthermore, reports suggest that the expression of androgen receptor (AR) increases with age in BC patients [35–37] and that the AR-positive cases show low pCR rates than the AR-negative cases [38]. Additionally, newer biomarkers may also affect these outcomes.

Moreover, von Waldenfels et al. have reported that prognosis worsens with age in BC patients [13]. However, their study observed significant differences in prognoses between patients aged ≥ 65 years and those aged 40–50 or 51–65 years, but no significant difference between patients aged ≥ 65 years and those aged < 40 years. Furthermore, studies reporting a higher pCR rate in younger patients did not observe a significant difference in prognosis in patients with TNBC [14]. In contrast, studies reported more than 10 years back suggest poor prognosis [39–41] and aggressive cellular properties in the younger BC patients [39, 42–44]. AR expression also affects prognosis and may contribute [38]. Additionally, with the advent of newer biological treatments, the number of clinical trials claiming prognosis to differ with age has decreased.

Here, when we studied TILs at all ages, we observed a correlation between TILs and clinicopathological factors, treatment effects, and prognosis similar to those reported previously. Moreover, our analysis suggests that younger BC patients had significantly higher TIL density than older BC patients. Additionally, age-related ORR and pCR rates differed in HER2-positive BC. Moreover, a pooled analysis for TNBC alone reported that the older patients had significantly lower TILs than the younger patients [45]. This result can be attributed to the decrease in host immunity due to aging, and to the inherent cellular characteristics of BC that vary with age.

However, this study has a limitation that the criteria for dividing patients into younger and older patients were not well-defined and that the clinicopathological factors other than TIL density differed with age. In addition, genetic predisposition, medications such as steroids, and lifestyle may also affect the immune microenvironment, but these factors could not be examined because this was a retrospective study. Furthermore, in this study, TILs were collectively examined, but they have various subclasses. As a typical example, CD8-positive cytotoxic T cells are reported to have a better prognosis as they are highly expressed [46–48], on the other hand, regulatory T cells, which were famous for being positive for FOXP3, were reported to be involved in poor prognosis [46]. PD-1 / PD-L1, which is also a target molecule in clinical treatment, might also affect TILs and prognosis [47, 49]. In addition, a study has reported that the host’s immune environment itself affects the pCR of preoperative chemotherapy [50]. In the future, it will be necessary to study immunohistochemical staining in our research as well. However, it was important to know the difference depending on the age in the evaluation of TILs by hematoxylin and eosin staining. And our study is considered to be the key study to show the reason why the therapeutic effect by age was different. The change with age in TME suggests that it may have influenced the therapeutic effect due to the characteristics of the host’s immune system, and the differences in cancer itself depending on the age. Additionally, in lung cancer, it has been reported that the therapeutic effect of the immune checkpoint inhibitors (ICIs) decreases in the older patients [51–53]. Therefore, age may also serve as an important clinical factor in deciding the course of treatment of BC patients with ICIs.

## Conclusions

The analysis presented in this study suggests that younger BC patients show significantly higher TIL density than older patients, along with differences in prognoses between the groups. Moreover, these differences may allow selection of better treatment modalities for the highly immunogenic subtypes of BC.

## Supplementary Information

**Additional file 1: Supplementary Figure S1.** Histopathological analysis of TIL density. The TIL density was calculated as average of infiltrating lymphocytes in the tumor stroma from five random fields, and graded as: a) 3 (>50%), b) 2 (10–50%), c) 1 (≤10%), and d) 0 (absent).

**Additional file 2: Supplementary Figure S2.** Comparison of disease-free survival (DFS) between high and low TIL density with varied BC subtypes. Kaplan-Meier DFS analysis has been indicated for patients grouped based on their BC subtype as: a) all cases, b) HR+HER2-, c) HR+HER2+, d) HER2-enriched, and e) TNBC. *P*-values in the figure indicate statistical significance for each comparison obtained using log-rank.

**Additional file 3: Supplementary Figure S3.** Comparison of overall survival (OS) between high and low TIL density with varied BC subtypes. Kaplan-Meier OS analysis has been indicated for patients grouped based on their BC subtype as: a) all cases, b) HR+HER2-, c) HR+HER2+, d) HER2-enriched, and e) TNBC. *P*-values in the figure indicate statistical significance for each comparison obtained using log-rank test.

**Additional file 4: Supplementary Table S1.** Difference in clinicopathological features due to TILs in all patients.

**Additional file 5: Supplementary Table S2.** Univariate and multivariate analysis with respect to DFS in all patients.

**Additional file 6: Supplementary Table S3.** Univariate and multivariate analysis with respect to OS in all patients.

## Data Availability

The datasets used and/or analyzed during the current study are available from the corresponding author on reasonable request.

## Abbreviations

AR: Androgen receptor
BC: Breast cancer
CIs: Confidence intervals
cCR: Clinical complete response
CNB: Core needle biopsy
CT: Computed tomography
cPD: Clinical progressive disease
cPR: Clinical partial response
cSD: Clinical stable disease
DFS: Disease-free survival
ER: Estrogen receptor
HER2: Human epidermal growth factor receptor 2
HR: Hormone receptor
ORR: Objective response rate
OS: Overall survival
pCR: Pathological complete response
PgR: Progesterone receptor
POC: Pre-operative chemotherapy
TN: Triple-negative
TILs: Tumor-infiltrating lymphocytes
TIME: Tumor microenvironment
US: Ultrasonography
VAB: Vacuum-assisted biopsy
