# Supplementary figures and images for: Differences in tumor-infiltrating lymphocyte density and prognostic factors for breast cancer by patient age
Source: World J Surg Oncol. 2022 Feb 17;20:38. doi: 10.1186/s12957-022-02513-5 (PMC8851811; doi:10.1186/s12957-022-02513-5)

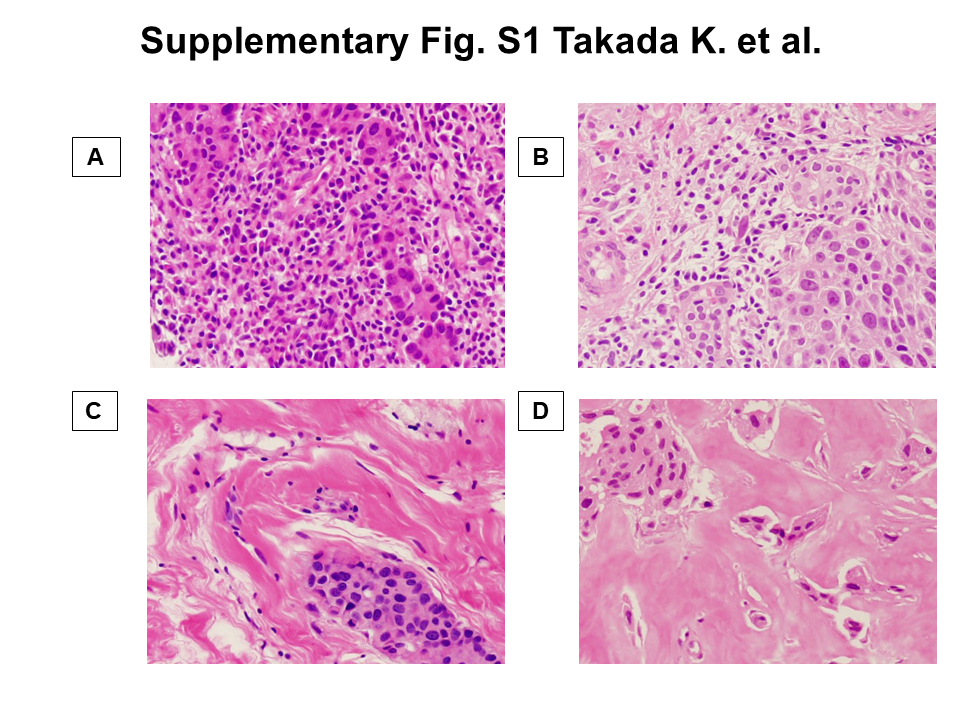

Supplement: Supplementary file 1 — Additional file 1: Supplementary Figure S1. Histopathological analysis of TIL density. The TIL density was calculated as average of infiltrating lymphocytes in the tumor stroma from five random fields, and graded as: a) 3 (>50%), b) 2 (10–50%), c) 1 (≤10%), and d) 0 (absent). [file 12957_2022_2513_MOESM1_ESM.tif]

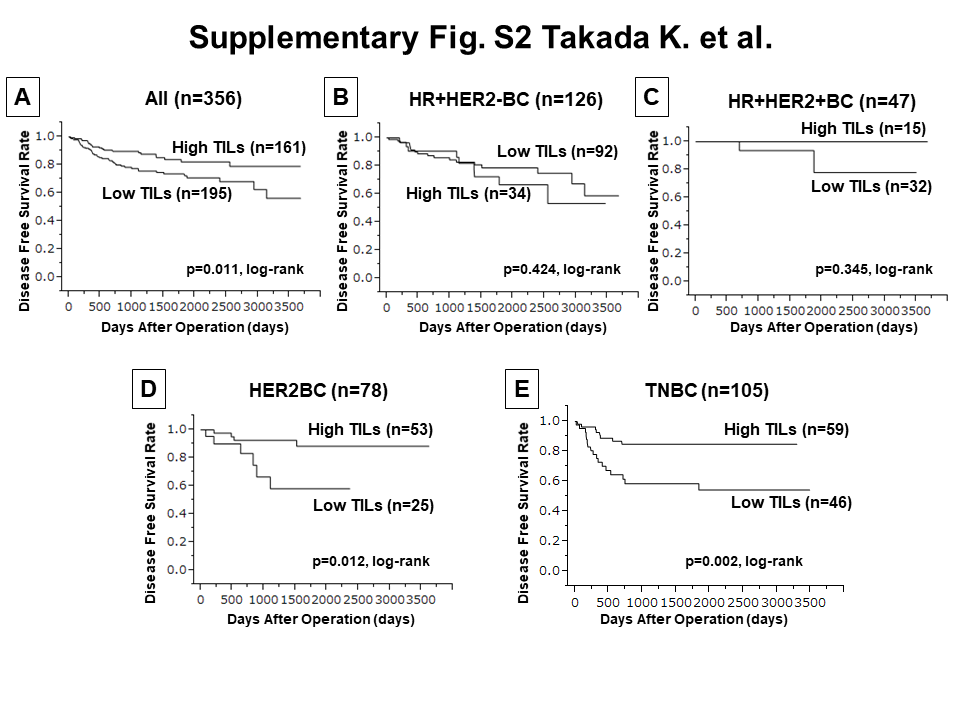

Supplement: Supplementary file 2 — Additional file 2: Supplementary Figure S2. Comparison of disease-free survival (DFS) between high and low TIL density with varied BC subtypes. Kaplan-Meier DFS analysis has been indicated for patients grouped based on their BC subtype as: a) all cases, b) HR+HER2-, c) HR+HER2+, d) HER2-enriched, and e) TNBC. P-values in the figure indicate statistical significance for each comparison obtained using log-rank. [file 12957_2022_2513_MOESM2_ESM.tif]

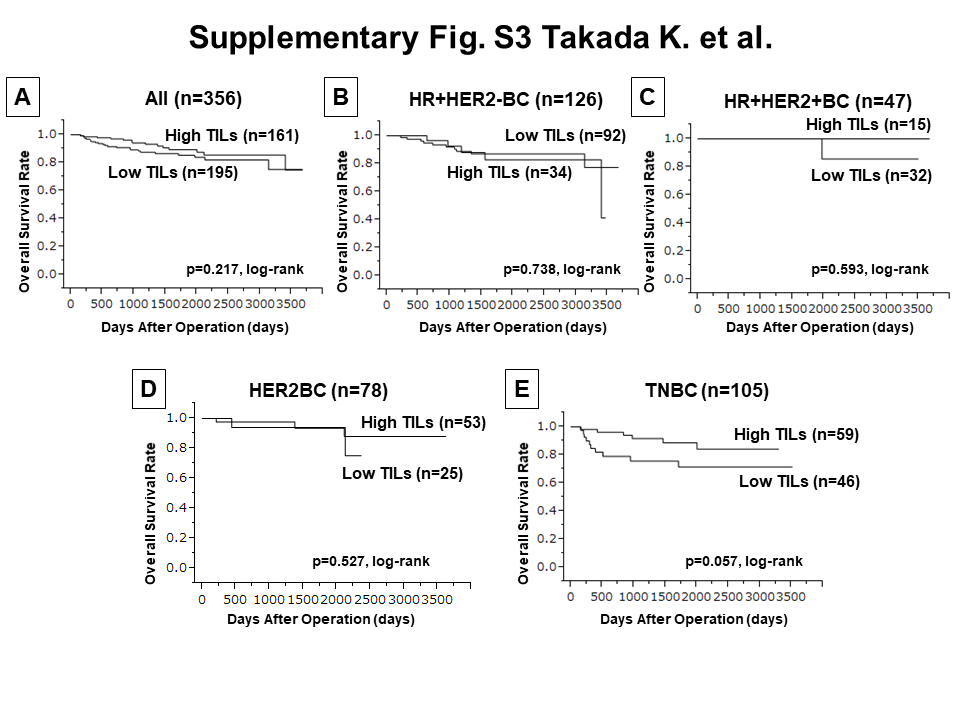

Supplement: Supplementary file 3 — Additional file 3: Supplementary Figure S3. Comparison of overall survival (OS) between high and low TIL density with varied BC subtypes. Kaplan-Meier OS analysis has been indicated for patients grouped based on their BC subtype as: a) all cases, b) HR+HER2-, c) HR+HER2+, d) HER2-enriched, and e) TNBC. P-values in the figure indicate statistical significance for each comparison obtained using log-rank test. [file 12957_2022_2513_MOESM3_ESM.tif]
