# Supplementary material for: Differences in tumor-infiltrating lymphocyte density and prognostic factors for breast cancer by patient age
Source: World J Surg Oncol. 2022 Feb 17;20:38. doi: 10.1186/s12957-022-02513-5 (PMC8851811; doi:10.1186/s12957-022-02513-5)
Supplement: Supplementary file 4 — Additional file 4: Supplementary Table S1. Difference in clinicopathological features due to TILs in all patients. [file 12957_2022_2513_MOESM4_ESM.docx]

**Supplementary Table S1. Difference in clinicopathological features due to TILs in all patients**

| Parameters | tumor- infiltrating lymphocytes (*n* = 356) | | |
| --- | --- | --- | --- |
|  | Low (*n* =195) | High (*n* = 161) | *p* value |
| Age (years old)  ≤ 45  > 45 | 31 (15.9%)  164 (84.1%) | 44 (27.3%)  117 (72.7%) | 0.008 |
| Tumor size (mm)  ≤ 20.0  > 20.0 | 35 (17.9%)  160 (82.1%) | 24 (14.9%)  137 (85.1%) | 0.442 |
| Skin infiltration  Negative  Positive | 152 (77.9%)  43 (22.1%) | 146 (90.7%)  15 (9.3%) | 0.001 |
| Lymph node status  Negative  Positive | 63 (32.3%)  132 (67.7%) | 58 (36.0%)  103 (64.0%) | 0.461 |
| Estrogen receptor  Negative  Positive | 73 (37.4%)  122 (62.6%) | 114 (70.8%)  47 (29.2%) | <0.001 |
| Progesterone receptor  Negative  Positive | 112 (57.4%)  83 (42.6%) | 130 (80.7%)  31 (19.3%) | <0.001 |
| Hormone receptor  Negative  Positive | 71 (36.4%)  124 (63.6%) | 112 (69.6%)  49 (30.4%) | <0.001 |
| HER2  Negative  Positive | 138 (70.8%)  57 (29.2%) | 93 (57.8%)  68 (42.2%) | 0.011 |
| Ki67  ≤14 %  >14 % | 81 (41.5%)  114 (58.5%) | 36 (22.4%)  125 (77.6%) | <0.001 |
| ORR  Non-Responders  Responders | 32 (16.4 %)  163 (83.6 %) | 8 (5.0%)  153 (95.0%) | 0.001 |
| Pathological response  Non-pCR  pCR | 156 (80.0%)  39 (20.0%) | 82 (50.9%)  79 (49.1%) | <0.001 |

TILs: tumor- infiltrating lymphocytes. HER: human epidermal growth factor receptor. ORR: objective response rate. CR: complete response.
