# Supplementary material for: Differences in tumor-infiltrating lymphocyte density and prognostic factors for breast cancer by patient age
Source: World J Surg Oncol. 2022 Feb 17;20:38. doi: 10.1186/s12957-022-02513-5 (PMC8851811; doi:10.1186/s12957-022-02513-5)
Supplement: Supplementary file 5 — Additional file 5: Supplementary Table S2. Univariate and multivariate analysis with respect to DFS in all patients. [file 12957_2022_2513_MOESM5_ESM.docx]

**Supplementary Table S2. Univariate and multivariate analysis with respect to DFS in all patients**

|  | Univarite analysis | | |  | Multivariate analysis | | |
| --- | --- | --- | --- | --- | --- | --- | --- |
| Parameters | Hazard ratio | 95% CI | *p* value |  | Hazard ratio | 95% CI | *p* value |
| Age at opetation (yr)  ≤ 45 vs > 45 | 0.927 | 0.533-1.710 | 0.797 |  |  |  |  |
| Tumor size (mm)  ≤ 20 vs > 20 | 1.227 | 0.621-2.789 | 0.578 |  |  |  |  |
| Skin infiltration  Negative vs Positive | 2.180 | 1.197-3.768 | 0.012 |  | 2.089 | 1.107-3.772 | 0.024 |
| Lymph node status  Negative vs Positive | 2.918 | 1.475-6.631 | 0.001 |  | 2.553 | 1.279-5.837 | 0.006 |
| Estrogen receptor  Negative vs Positive | 0.812 | 0.492-1.327 | 0.406 |  |  |  |  |
| Progesterone receptor  Negative vs Positive | 0.940 | 0.548-1.565 | 0.817 |  |  |  |  |
| Hormone receptor  Negative vs Positive | 0.814 | 0.495-1.330 | 0.412 |  |  |  |  |
| HER2  Negative vs Positive | 0.498 | 0.254-0.901 | 0.020 |  | 0.640 | 0.303-1.280 | 0.212 |
| Intrinsic subtype  Not TNBC vs TNBC | 1.574 | 0.941-2.584 | 0.083 |  | 1.704 | 0.937-3.081 | 0.080 |
| Ki67  ≤14 % vs >14 % | 1.577 | 0.930-2.793 | 0.092 |  | 2.095 | 1.205-3.790 | 0.008 |
| Objective response rate  Non-Responders vs Responders | 0.247 | 0.145-0.439 | <0.001 |  | 0.286 | 0.158-0.532 | <0.001 |
| Pathological response  Non-pCR vs pCR | 0.315 | 0.151-0.591 | <0.001 |  | 0.391 | 0.180-0.781 | 0.007 |
| TILs  Low vs High | 0.512 | 0.298-0.852 | 0.010 |  | 0.699 | 0.382-1.247 | 0.227 |

DFS: Disease-free survival. CI: confidence intervals. HER: human epidermal growth factor receptor. pCR: pathological complete response. TILs: tumor- infiltrating lymphocytes.
