# Supplementary material for: Differences in tumor-infiltrating lymphocyte density and prognostic factors for breast cancer by patient age
Source: World J Surg Oncol. 2022 Feb 17;20:38. doi: 10.1186/s12957-022-02513-5 (PMC8851811; doi:10.1186/s12957-022-02513-5)
Supplement: Supplementary file 6 — Additional file 6: Supplementary Table S3. Univariate and multivariate analysis with respect to OS in all patients. [file 12957_2022_2513_MOESM6_ESM.docx]

**Supplementary Table S3. Univariate and multivariate analysis with respect to OS in all patients**

|  | Univarite analysis | | |  | Multivariate analysis | | |
| --- | --- | --- | --- | --- | --- | --- | --- |
| Parameters | Hazard ratio | 95% CI | *p* value |  | Hazard ratio | 95% CI | *p* value |
| Age at opetation (yr)  ≤ 45 vs > 45 | 0.689 | 0.349-1.456 | 0.314 |  |  |  |  |
| Tumor size (mm)  ≤ 20 vs > 20 | 1.450 | 0.595-5.027 | 0.422 |  |  |  |  |
| Skin infiltration  Negative vs Positive | 3.681 | 1.803-7.205 | 0.001 |  | 3.695 | 1.767-7.454 | 0.001 |
| Lymph node status  Negative vs Positive | 3.034 | 1.205-10.185 | 0.016 |  | 2.467 | 0.964-8.360 | 0.061 |
| Estrogen receptor  Negative vs Positive | 0.744 | 0.378-1.423 | 0.373 |  |  |  |  |
| Progesterone receptor  Negative vs Positive | 0.826 | 0.392-1.630 | 0.592 |  |  |  |  |
| Hormone receptor  Negative vs Positive | 0.785 | 0.403-1.500 | 0.465 |  |  |  |  |
| HER2  Negative vs Positive | 0.422 | 0.158-0.945 | 0.035 |  | 0.423 | 0.144-1.091 | 0.076 |
| Intrinsic subtype  Not TNBC vs TNBC | 1.824 | 0.936-3.488 | 0.077 |  | 1.693 | 0.809-3.549 | 0.161 |
| Ki67  ≤14 % vs >14 % | 1.478 | 0.749-3.120 | 0.267 |  |  |  |  |
| Objective response rate  Non-Responders vs Responders | 0.224 | 0.115-0.463 | <0.001 |  | 0.211 | 0.103-0.453 | <0.001 |
| Pathological response  Non-pCR vs pCR | 0.335 | 0.126-0.749 | 0.006 |  | 0.471 | 0.169-1.134 | 0.096 |
| TILs  Low vs High | 0.660 | 0.331-1.268 | 0.214 |  |  |  |  |

OS: Overall survival. CI: confidence intervals. HER: human epidermal growth factor receptor. pCR: pathological complete response. TILs: tumor- infiltrating lymphocytes.
